# IFNγ-mediated suppression of alternative NF-κB in tumor-resident myeloid cells promotes selective recruitment of cytotoxic but not regulatory T cells

**DOI:** 10.3389/fimmu.2025.1681777

**Published:** 2025-10-09

**Authors:** Adam Brinkman, Ravikumar Muthuswamy, Bowen Dong, Robert P. Edwards, Pawel Kalinski

**Affiliations:** ^1^ Department of Immunology, Roswell Park Comprehensive Cancer Center, Buffalo, NY, United States; ^2^ Department of Surgery, University of Pittsburgh Hillman Cancer Center, Pittsburgh, PA, United States; ^3^ Department of OBGYN, Magee Women’s Hospital and Research Institute, Pittsburgh, PA, United States; ^4^ Department of Medicine, Roswell Park Comprehensive Cancer Center, Buffalo, NY, United States

**Keywords:** chemokines, CTLs, Tregs (regulatory T cells), interferon gamma (IFNγ), alternative NF-κB, myeloid cells, tumor micro environment (TME)

## Abstract

Immunotherapy is currently effective in less than half of patients with solid tumors, and most responders develop secondary progression. High infiltration of the tumor microenvironment (TME) with CD8^+^ cytotoxic T cells (CTLs) and low infiltration with regulatory T cells (Treg) predicts the patients’ responses to immunotherapy and long-term outcomes. To identify the mechanisms regulating long-term stability of CTL infiltration, we analyzed the impact of CTL-produced cytokines on the TME by co-culturing patient-isolated ascites cells with activated T cells. Unexpectedly, we observed that activated CTLs selectively induce cytotoxic T cell-attracting chemokines but not chemokines that attract T regulatory cells in ovarian cancer TME and tumor-associated myeloid cells, resulting in recruitment of additional CTLs without Tregs. This selectivity resulted from the unique dependence of CCL22 induction on both canonical and alternative NF-κB and the suppression of alternative NF-κB signaling by T cell-released IFNγ. Our data demonstrate that T cell-produced IFNγ suppresses alternative NF-κB signaling in TME-associated myeloid cells, allowing for the induction of CTL-attracting chemokines with the concomitant suppression of Treg-attracting CCL22. These novel functions of IFNγ and activated T cells in regulating the balance between canonical and alternative NF-κB signaling in myeloid cells provide new opportunities to enhance and stabilize the selective CTL influx in the TME.

## Introduction

Despite recent progress in cancer immunotherapy, less than half of patients with solid tumors respond to current immunotherapies with most of them exhibiting only transient responses (1, 2). One factor limiting the effectiveness of immunotherapy is the composition of the tumor microenvironment (TME) (3). High frequencies of Granzyme B^+^ CD8^+^ cytotoxic T lymphocytes (CTLs) in the TME are necessary for effective antitumor immunity and are associated with improved prognoses and responses to immunotherapy in many cancer types (4, 5). In contrast, TME infiltration with T regulatory cells (Tregs) is associated with poor prognosis and diminished response to immunotherapy and other cancer treatments (6, 7). Although many immunotherapies and chemotherapies have been shown to promote attraction of CTLs, the durability and selectivity of their effects in promoting CTL but not Treg attraction remains limited (8, 9).

Immune cell trafficking into the TME is controlled by chemokines (8, 10). CTLs express high levels of chemokine receptors CCR5 and CXCR3 which drive their migration towards tissues expressing the cognate ligands CCL5/RANTES and CXCL9/10/11 (11). Accordingly, tumors that express high levels of CCL5 and CXCL10 are associated with elevated numbers of antitumor CTLs (12, 13). In contrast, tumors with high production of CCL22 recruit immunosuppressive CCR4^+^ Tregs (6, 12, 14, 15). These considerations led us to develop a chemokine modulatory (CKM) regimen combining TLR3 agonists (double-stranded RNA species: poly-I:C or rintatolimod) and type-1 Interferons (IFNα), which has recently shown safety and ability to selectively reprogram the chemokine production in the TME of cancer patients and increase CTL infiltration without Tregs or MDSCs (12, 16–23). However, because such intratumoral effects have been transient (18, 21, 22), there is a need to identify mechanisms which affect the magnitude and duration of chemokine production and can be targeted to stabilize the treatment-induced CTL accumulation and effector function.

The canonical and alternative (non-canonical) NF-κB signaling pathways (**Supplementary Figure 1**) are critical for multiple aspects of cancer cell and TME biology, including chemokine production, resulting in multiple efforts to target this pathway in cancer therapy (24, 25). Both pathways involve pre-formed heterodimers that remain in the cytoplasm until triggered, after which their phosphorylation induces nuclear translocation and transcription of target genes (26). Canonical NF-κB signaling is critical for the induction of both tumor-promoting chemokines, such as CCL22, and the chemokines involved in tumor rejection, such as CCL5 and CXCL10 (12, 17, 27, 28). We have observed that the chemokine modulatory effects of CKM depend on canonical NF-κB signaling and selectively target tumor tissues over healthy tissues due to the hyperactivation of the canonical NF-κB pathway in the stromal and myeloid cells of the TME (12, 17). In contrast, the alternative NF-κB pathway is known to be involved in the induction of homeostatic chemokines such as CCL19, CCL21, CXCL12 and CXCL13, and the suppressive CCL22, but its role in the attraction of effector cells remains unclear (29–31).

Several reports indicate only transient effector function of CTLs in the TME (2, 32–34), highlighting a need for targeted therapeutics that result in durable CTL influx and function. Upon their activation, CTLs release effector molecules that induce secondary effects in the TME which can either support or diminish antitumor immunity (35). For example, activated CTLs release TNFα and IFNγ that can induce dendritic cell (DC) maturation to support the generation of type-1 antitumor immunity (36), and can suppress TGF-β signaling (37). However, these same mediators also enhance cyclooxygenase-2 (COX2)/prostaglandin E2 (PGE2) signaling in MDSCs that self-limits antitumor immunity (38). Since the tumor immune composition influences what secondary effects can accumulate, we tested if activated T cells possess the ability to remodel the TME and induce recruitment of additional immune cells to bolster or diminish antitumor immunity.

## Materials and methods

### Human samples

Ovarian cancer ascites samples were collected during routine procedures under the University of Pittsburgh IRB-approved tissue banking protocol UPCI 07-058 (Prognostic Marker: Acquisition of Blood Samples and Tissue for Research Purposes; Gyn-Onc # 22-096). Ascites fluid cells were isolated by centrifugation and cultured for subsequent analyses in AIM-V medium (Gibco #12055-091). Human peripheral blood cones were obtained from healthy adult volunteers (as a product of platelet collection) under the Roswell Park Comprehensive Cancer Center IRB-approved protocol 163222. PBMCs, monocytes, and lymphocytes were isolated as described below and stored in liquid nitrogen until ready for experiments.

### Generation of effector T cells

Naïve CD8^+^ T cells were purified from peripheral blood mononuclear cells (PBMCs) of healthy donors using an EasySep naïve CD8^+^ T cell enrichment kit (StemCell Technologies, #17968). Isolated naïve CD8^+^ T cells were stimulated with CD3/CD28 microbeads (Gibco #11131D) and IL-12p70 (20 ng/ml, Peprotech #200-12-50UG) for 7 days. On day 7, effector CD8^+^ T cells were harvested, cells were counted and adjusted to 1,000,000 cells/mL, reactivated, and used for subsequent assays.

### Generation of monocyte-derived macrophages

PBMCs were isolated from healthy donor blood through lymphocyte separation medium (Ficoll) as previously described (12, 27). Monocytes were isolated from light fraction of PBMCs through Percoll density gradient centrifugation. Monocytes were cultured at 500,000 per well in 24-well plates (Corning #353047) for 6 days in IMDM media (Gibco #12440-053) supplemented with 10% FBS (Gibco #10082-147) and 1000 IU/mL recombinant human GM-CSF (Miltenyi #130-093-868) to generate macrophages. On day 3 of cultures, half of the media was replenished with fresh IMDM 10% FBS with double concentration of GM-CSF. Macrophages were harvested by incubating wells in 0.5mL TrypLE Select (Gibco #12563-029) at 37 degrees Celsius for 30 minutes followed by gentle scraping. Macrophages were collected, washed, and re-plated in IMDM 10% FBS in the appropriate plates for the indicated experiments.

### Treatment of cell cultures with cytokines and inhibitors

Ovarian ascites cells or macrophages were cultured 100,000 per well in a 96-well plate (Corning #3599) then stimulated with either 50 ng/mL TNFα (Miltenyi #130-094-562) and/or 1000 IU/mL IFNγ (Miltenyi #130-096-484) for 24 hours. Supernatants and total RNA were collected as described below. When indicated, cells were pre-treated for 2 hours with small molecule inhibitors for canonical NF-κB (JSH-23 30µM, Selleckchem #S7351) or alternative NF-κB (NIK-SMI1 2µM, MedChemExpress #HY-112433) before treatment. Treatment with AZD5582 (5nM Selleckchem #S7362) was used to activate alternative NF-κB signaling as a control.

### Co-culture of CD8^+^ T cells with ovarian ascites or macrophages

Co-cultures with T cells and either ovarian ascites cells or macrophages were done as previously described (16, 17, 27, 38, 39). Briefly, a total of 500,000 isolated ovarian ascites cells or macrophages were cultured overnight. On the next day, plates were spun down at 600 RPM for 5 minutes, media from the wells were removed and replaced with either 1mL of media or 1mL of media containing 100,000 re-stimulated CD8^+^ T cells and cultured for 24 hours. For neutralization of T cell-derived TNFα and IFNγ, 10µg/mL of blocking antibodies against TNFα (BD Biosciences #554508, RRID: AB_395441) and IFNγ (BD Biosciences Cat# 554698, RRID: AB_395516) were added to cultures. For detection of intracellular chemokines, monensin (2µM, Bio-Rad #BUF074) was added during the last 5 hours to block chemokine secretion.

### Quantitative PCR

Total RNA was extracted using the RNeasy kit (Qiagen #74104). Synthesis of cDNA was performed according to the qScript protocol (QuantaBio #95047) using 250ng RNA per sample and a Bio-Rad T100 Thermal Cycler. All cDNA was diluted 5 times before analysis by qPCR. RT-PCR was performed on a Bio-Rad CFX96 Real-Time PCR Detection System (RRID: SCR_018064) using 4µL diluted cDNA per reaction and iTaq universal probe supermix protocol (Bio-Rad #1725134). Gene expression was determined using endogenous HPRT as a control. The following primers were purchased from ThermoFisher and used for analysis: HPRT (Life Technologies 4325801); CCL5 (TaqMan Hs00174575_m1); CXCL10 (TaqMan Hs00171042_m1); CCL22 (TaqMan Hs01574247_m1).

### ELISA

Protein concentrations in culture supernatants were measured by sandwich ELISA. Primary and biotinylated detection antibody pairs were purchased from R&D Systems. High binding plates (Corning #3361) were coated overnight with primary antibody (concentration is target-dependent according to manufacturer protocol), followed by washing and blocking with DPBS 2% BSA (MP Biomedicals #160069) for 1 hour. Samples were added to plate and incubated for 2 hours, then biotinylated detection antibodies were added for 1 hour, followed by a 30-minute incubation with Streptavidin-HRP conjugate (R&D Systems #DY998). All antibodies and HRP conjugates were diluted in blocking buffer at manufacturer-recommended concentrations. Protein levels were detected by adding 100µL TMB substrate solution (ThermoScientific #34029), then reactions were stopped with equal volume 2N sulfuric acid. Absorbance at 450nm was recorded using a BioTek Epoch microplate reader (Agilent, California, RRID: SCR_019741) and analyzed using Gen5 software (RRID: SCR_017317).

### Flow and imaging cytometry

Cells were fixed with 4% paraformaldehyde (ThermoFisher #J19943-K2) for 10 minutes, washed with DPBS, then kept at 4 degrees C overnight in flow buffer (DPBS with 2% BSA and 0.02% sodium azide from Millipore Sigma with 2mM EDTA from Invitrogen) to block Fc receptors. Cells were surface stained with designated antibodies in flow buffer for 30 minutes, washed, then permeabilized using either 0.1% Triton-X (ThermoScientific #A16046.AE, for macrophages and ascites cells) or 1x FoxP3 permeabilization buffer (BioLegend #421002, for migrated T cells) containing appropriate dilutions of antibodies for 45 minutes. When indicated, cells were nuclear counterstained with a 1µM DRAQ5 solution (ThermoFisher #650880-92) for 3 minutes before the final wash. Cell viability experiments were carried out with a 1mM DAPI solution (Millipore Sigma D9542) and determined by DAPI positivity. Flow cytometry samples were analyzed on a BD Fortessa cytometer by collecting at least 10,000 single cells. Imaging cytometry was performed using a Cytek Amnis Image Stream Mk II cytometer and collected at least 3000 single cells with a Gradient RMS greater than 50 (in focus). The following antibodies were purchased for these studies: NF-κB p65/RelA (Santa Cruz Biotechnology Cat# sc-8008, RRID: AB_628017), NF-κB p52/p100 (Santa Cruz Biotechnology Cat# sc-7386, RRID: AB_2267131), CCL5 (Bd Biosciences Cat# 564754, RRID: AB_2738932), CXCL10 (BD Biosciences Cat# 555049, RRID: AB_395670), CD8 (BD Biosciences Cat# 563823, RRID: AB_2687487), CD33 (BD Biosciences Cat# 551378, RRID: AB_398502), CD4 (BD Biosciences Cat# 555347, RRID: AB_395752), Granzyme B (BD Biosciences Cat# 560211, RRID: AB_1645488), FoxP3 (BioLegend Cat# 320124, RRID: AB_2565972), and CD326 (BioLegend Cat# 324208, RRID: AB_756082). Flow cytometry data were analyzed using FloJo software (FloJo, LLC). Image Stream data were analyzed using IDEAS software (Amnis).

### Chemotaxis 

Chemotaxis assays were performed as previously described (16, 17, 28, 39, 40). Briefly, in a 24 trans-well plate with 5µm membrane pore size (Corning #3421), 500µL of culture supernatants were added to the bottom chambers and 200µL containing 200,000 T cells (either CD4^+^ or CD8^+^ isolated as described above) were added to the top chambers. After 60 minutes, bottom chambers were collected and analyzed for CTLs (CD8^+^ GzmB^+^) or Tregs (CD4^+^ FoxP3^+^) with flow cytometry. Total numbers were quantified by using CountBright Plus Ready Tubes (Invitrogen #C40000).

### Statistical analysis

All statistics were performed using GraphPad Prism 10 software (RRID: SCR_002798). Comparisons between groups were tested using one-way ANOVA with Tukey’s correction. The values of P <0.05 were considered as significant (ns = not significant; *p < 0.05; **p < 0.005; ***p < 0.0005; ****p < 0.0001). Each experiment was performed in triplicate unless indicated otherwise. All experiments were successfully reproduced at least three times with different donors/patients. Data shown represent the replicates from the same donor as mean +/- SEM. Due to the nature of the study, no randomization, blinding, or power analysis was required.

### Data availability

All data generated in this study are available within the article and its **Supplementary Data Files** or from the corresponding author (P. Kalinski) upon reasonable request.

## Results

### Activated CTLs selectively induce CTL-attracting chemokines in ovarian cancer ascites without inducing Treg attractants

**Figure 1:**
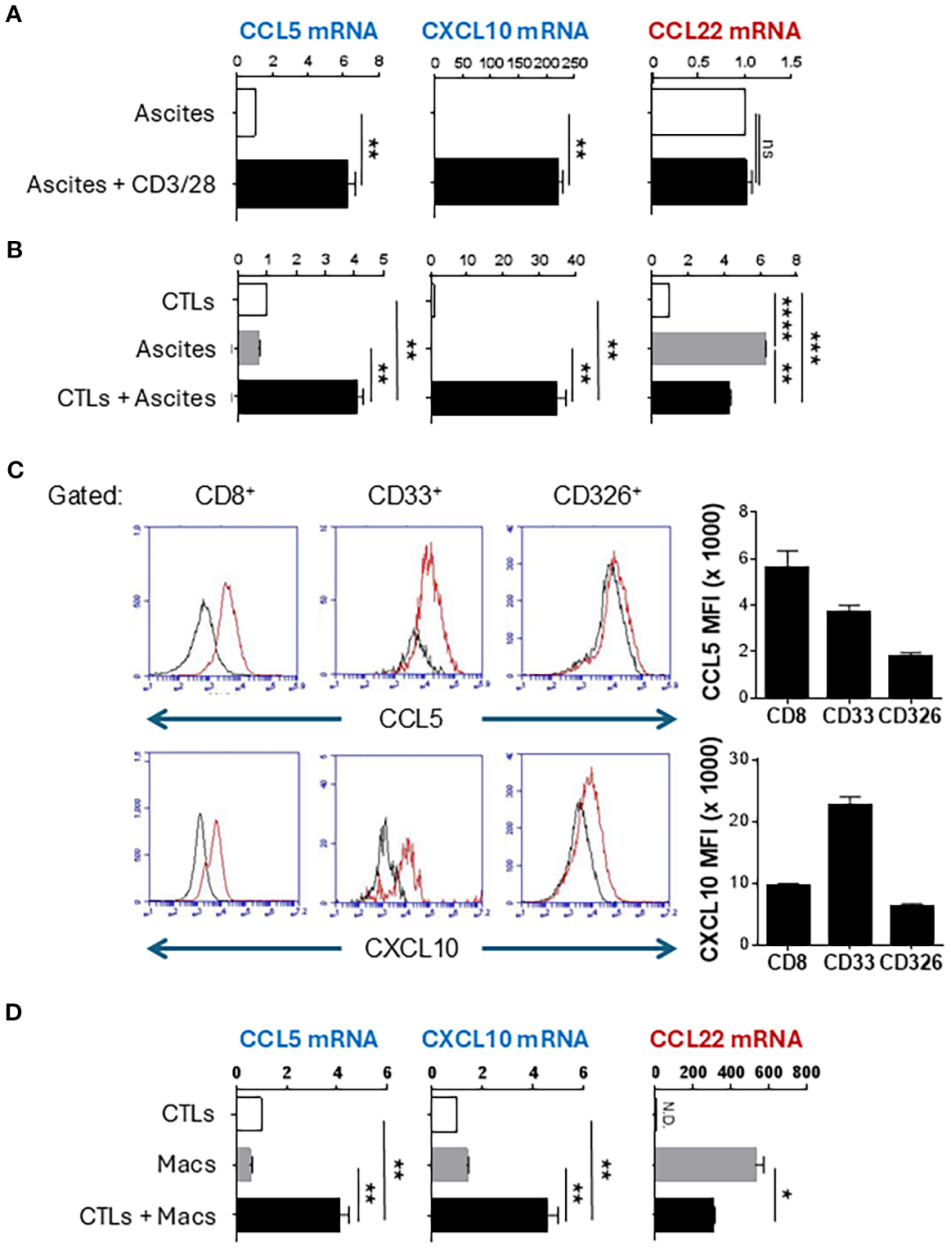
Activated CTLs selectively induce CTL-attracting chemokines in human ovarian cancer-associated myeloid cells and cultured macrophages. **(A)** Patient-isolated ovarian ascites cells were stimulated with CD3/CD28 activating beads for 24 hours. Chemokine expression was measured by TaqMan qRT-PCR. **(B)** Patient-isolated ovarian ascites cells were co-cultured with activated *ex vivo*-expanded CTLs for 24 hours, then chemokine production was measured by TaqMan qRT-PCR. **(C)** Co-cultures of CTLs and ascites cells were surface stained for indicated markers followed by intracellular staining for chemokines. Gray parameter represents fluorescence minus one (FMO). Bar graphs represent median fluorescence intensity of chemokine expression in the CD8+, CD33+, or CD326+ populations (mean +/- SEM, n=3). **(D)** Monocyte-derived macrophages (Macs) were co-cultured with activated CTLs for 24 hours followed by chemokine analysis by TaqMan qRT-PCR. TaqMan data are reported as Expression Fold Change (2^-ddCt^) normalized to either untreated ascites **(A)** or to CTLs **(B, D)** to eliminate the background chemokine expression by T cells. All data in this figure are mean +/- SEM of triplicate cultures from the same patient/donor and represent one of three independent experiments with similar results from different patients/donors. ns, not significant; *p < 0.05; **p < 0.005; ***p < 0.0005; ****p < 0.0001.

Malignant ovarian ascites and the associated ascites cells offer a unique opportunity to study local immune modulation because they involve inactive/dysfunctional CD4^+^ and CD8^+^ T cells which lack effector functions and CCL22-dependent accumulation of suppressive Tregs (6, 13, 17, 41). To test the impact of activated T cells on chemokine production within the TME, we collected total ascites cells from ovarian cancer patients undergoing cyto-reductive surgery and stimulated them with CD3/28- beads to activate the ascites-associated T cells. Interestingly, activation of such endogenous T cells was highly effective in inducing CTL-attracting chemokines CCL5 and CXCL10 (**Figure 1A**). Since local activation of T cells in the ovarian TME can induce COX2/PGE2 signaling and immunosuppression (38), we also examined the expression of CCL22 which we previously identified as a COX2-PGE2-dependent Treg attractant (12, 28). Unexpectedly, the induction of CTL-attracting chemokines in response to CD3/CD28 activation was not accompanied by enhancement of CCL22, indicating that T cell-activating signals can allow for the selective induction of desirable chemokines in ovarian TME (**Figure 1A**).

To test if CTLs are sufficient to reprogram the ovarian TME, we expanded healthy blood-isolated CD8^+^ T cells *ex vivo* for 7 days, using CD3/CD28 beads and IL-12p70 to generate effector CTLs (16). After overnight activation with CD3/28 beads, these “exogenous” CTLs were cultured with isolated ovarian cancer ascites cells. Such activated CTLs strongly enhanced the production of CCL5 and CXCL10 (**Figures 1B**, **Supplementary Figure S2A**). Strikingly, these pre-activated CTLs reduced CCL22 expression in ovarian cancer ascites, compared to the levels spontaneously produced by the ascites cells (**Figure 1B**). These data demonstrate that activated CTLs, both resident and exogenous, can reprogram the ovarian TME to selectively induce CTL-attracting chemokines, but not suppressive chemokines.

### Myeloid cells are the major source of CCL5 and CXCL10 induced in the TME by activated CTLs

To confirm that activated T cells were indeed the inducers of CCL5 and CXCL10 in other ascites cells (rather than being the only source themselves), we stained the co-cultures for intracellular CCL5 and CXCL10 and surface markers of CTLs (CD8), myeloid cells (CD33), and epithelial cells (EpCam, CD326). As shown in **Figure 1C**, CD33^+^ myeloid cells constituted the dominant source of CXCL10 while CCL5 was produced by both myeloid cells and CTLs. To validate the key role of myeloid cells in CTL-induced chemokine production, monocyte-derived macrophages were cultured with *ex vivo*-expanded CTLs, which revealed similar increases in CCL5 and CXCL10 expression, without increases in CCL22 (**Figure 1D**, **Supplementary Figure S2B**).

### CTL-derived TNFα and IFNγ synergize in the selective induction of CTL-attracting chemokines

**Figure 2:**
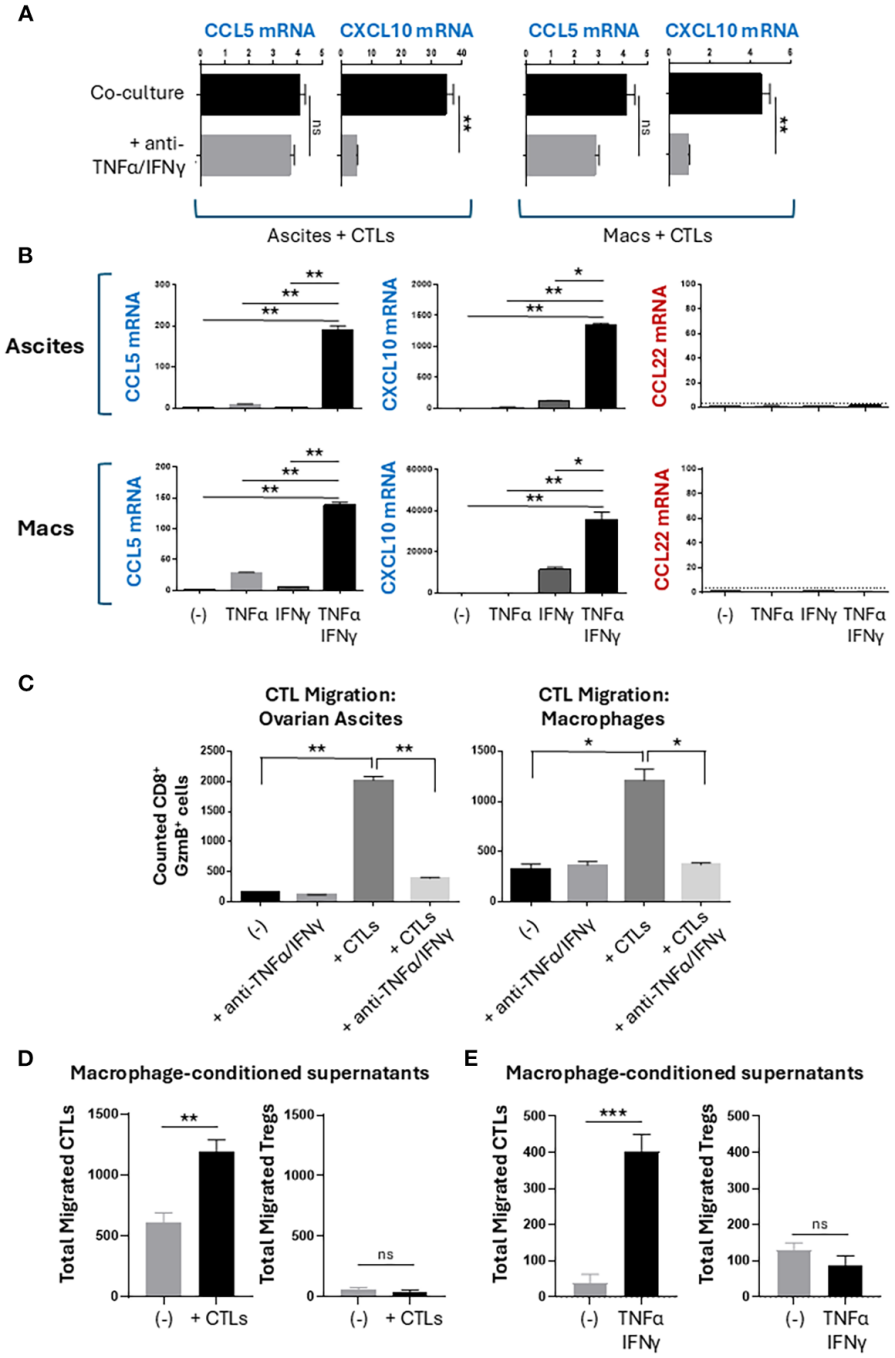
CTL-produced TNFα and IFNγ promote selective recruitment of CTLs but not Tregs. **(A, B)** Chemokine expression in 24-hour cultures. Data shown as relative mRNA levels (2^-dCt^) normalized for HPRT. **(C-E)** Migration of CTLs and Tregs. Supernatants from 24-hour cultures of either CTL-exposed ascites cells (C-left), CTL-exposed macrophages (C-right and **(D)**), or TNFα/IFNγ-exposed macrophages **(E)** were placed into the bottom chambers of a Transwell assay plate. Fresh CTLs or CD4^+^ T cells were placed into the top chambers, and after 60 minutes, the bottom chambers were collected and analyzed for migrated T cells. CD8^+^ GzmB^+^ CTLs or CD4^+^ FoxP3^+^ Tregs were counted using flow cytometry. All data are shown as mean +/- SEM of triplicate cultures from the same donor/patient and represents one of three independent experiments with similar results from different patients/donors. Mean background migration towards control media was subtracted from all conditions shown in panels **(C–E)**. ns, not significant; *p < 0.05; **p < 0.005; ***p < 0.0005.

Because CTL-derived TNFα and IFNγ contribute to the undesirable enhancement of MDSC suppressive function (38) but also to the desirable induction of DC maturation (36), we analyzed their roles in the CTL-mediated chemokine reprogramming of the TME. Neutralization of TNFα and IFNγ abrogated the induction of CXCL10 in co-cultures of CTLs with ovarian ascites cells or monocyte-derived macrophages (**Figures 2A**). The production of CCL5 was not significantly decreased by the TNFα/IFNγ blockade, which is consistent with T cells being the main source of CCL5 rather than T cell-activated myeloid cells (**Figure 1C**). To validate these causative roles, we treated the ascites cells or macrophages with recombinant human TNFα, IFNγ, or their combination. While single treatments with either TNFα or IFNγ did not or only marginally induced CCL5 and CXCL10, their combination synergistically induced CCL5 and CXCL10 in both ascites and macrophages (**Figures 2B**). In accordance with the previous data, neither of these factors induced expression of CCL22 in the ascites cells nor macrophages.

### TNFα and IFNγ-producing CTLs promote selective recruitment of CTLs, but not Tregs

To test the migratory capacity of TNFα/IFNγ-induced chemokine modulation, we used a Transwell migration system to measure migration of CTLs and Tregs towards conditioned supernatants (**Supplementary Figure S3A**) (16). We observed a strong increase in the numbers of CTLs that migrated towards conditioned supernatants of ovarian ascites or macrophages exposed to activated CTLs (**Figure 2C**). Increased CTL migration was dependent on the production of TNFα and IFNγ by the activated CTLs during co-culture. Because CCL22 is a known attractant of CCR4^+^ Tregs (6, 28) (**Supplementary Figure S3B**), we tested the impact of CCL22 modulation by activated CTLs. We observed that macrophages cultured with activated CTLs or stimulated with recombinant TNFα and IFNγ recruited additional CTLs but not Tregs (**Figures 2D, E**). Together, these data demonstrate a selective modulation of T cell recruitment to favor CTL but not Treg recruitment.

### Unique requirement for alternative NF-κB signaling in CCL22 induction

**Figure 3:**
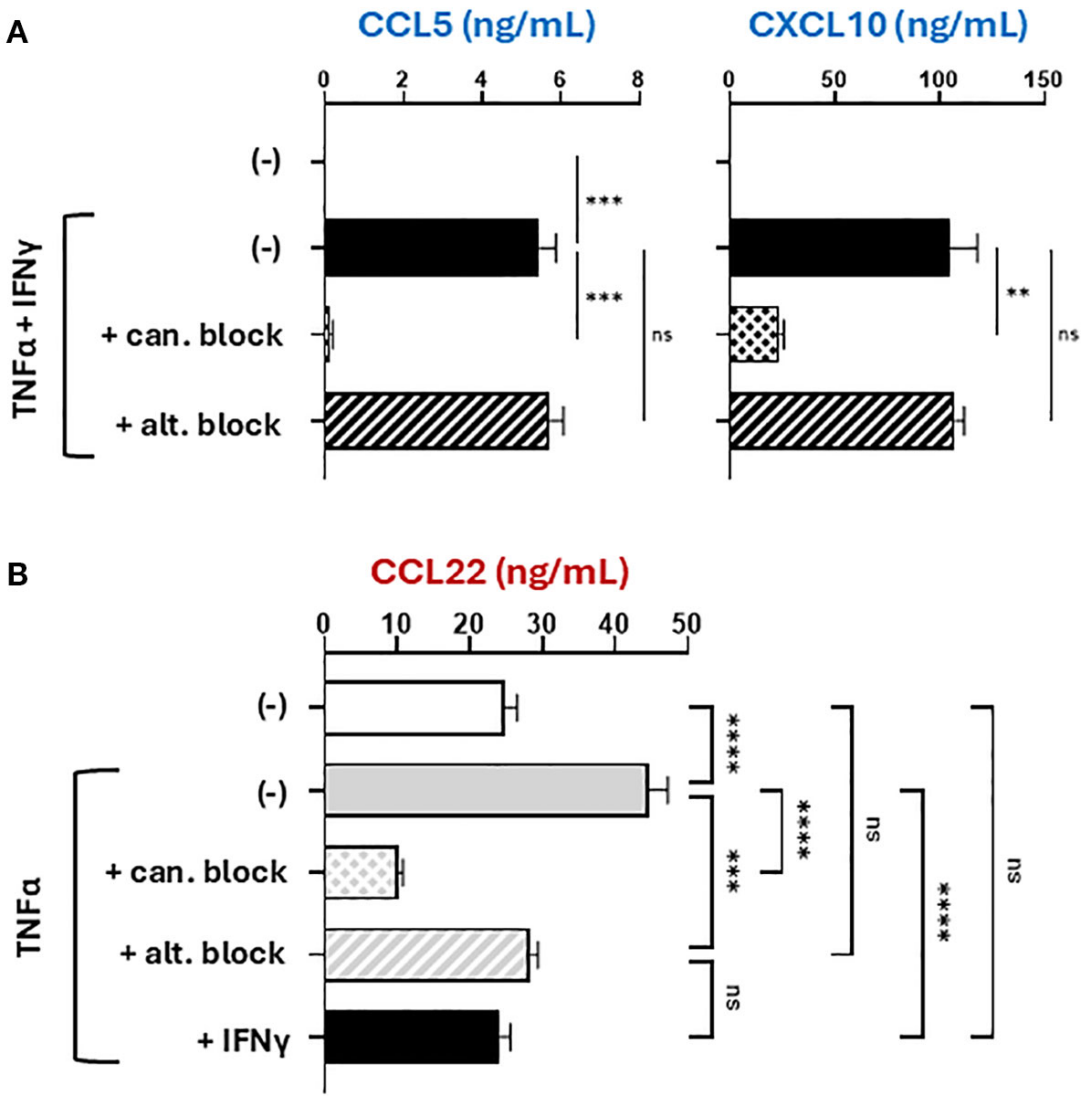
The induction of CCL22, but not CTL attractants, uniquely depends on both canonical and alternative NF-κB signaling. Macrophages were stimulated with recombinant human TNFα and/or IFNγ for 24 hours in the absence or presence of selective NF-κB inhibitors (given 2 hours prior to stimulation). 24-hour culture supernatants were analyzed by ELISA for CTL-attractants **(A)** or Treg attractants **(B)**. All data shown are mean +/- SEM of triplicate cultures from the same donor representing one of three independent experiments with different donors. ns, not significant; **p < 0.005; ***p < 0.0005; ****p < 0.0001.

Prompted by the observations that CCL22 can be induced by either canonical or alternative NF-κB signaling (17, 29, 42–44), we compared the roles of the two pathways in the regulation of CTL versus Treg attractants by TNFα and IFNγ, using small molecule inhibitors of either canonical NF-κB (JSH-23) or alternative NF-κB (NIK-SMI1) (45, 46). The specificity and selectivity of action of these inhibitors was validated by measuring nuclear translocation of NF-κB proteins with imaging cytometry (47) and cell viability (**Supplementary Figures S4A–C**). As expected, blockade of canonical NF-κB signaling abrogated the induction of all chemokines tested: CCL5, CXCL10, and CCL22 (**Figures 3A, B**). In contrast, alternative NF-κB blockade did not affect the production of CCL5 or CXCL10, showing that the induction of CTL-attracting chemokines requires only canonical but not alternative NF-κB signaling (**Figure 3A**). Simultaneously, alternative NF-κB blockade prevented the induction of CCL22, demonstrating that CCL22 induction requires both canonical and alternative NF-κB signaling (**Figure 3B**, **Supplementary Figure S4D**).

### IFNγ suppresses alternative NF-κB signaling to inhibit CCL22 production

We previously demonstrated that CCL22 induction in myeloid cells requires canonical NF-κB and COX2/PGE2 signaling (17) and confirmed these results in the current system (**Supplementary Figure S5A**). Since canonical NF-κB signaling was functional as evidenced by the enhanced production of CCL5 and CXCL10 by CTL-exposed or TNFα/IFNγ-treated macrophages, we tested if the reduced CCL22 production was due to a loss of COX2 signaling. In contrast to this possibility, both the expression of COX2 and a COX2-regulated gene, indoleamine 2,3-dioxygenase 1 (IDO1), increased in macrophages treated with TNFα, IFNγ, and especially in their combination, suggesting the loss of CCL22 production did not result from disrupted COX2 (**Supplementary Figure S5B**). Since these results suggested a regulatory relationship between COX2 and alternative NF-κB signaling, we tested if COX2/PGE2 signaling induces alternative NF-κB. Indeed, exposure to exogenous PGE2 upregulated nuclear p52 levels compared to baseline (**Supplementary Figure S5C**), indicating that COX2/PGE2 signaling induces CCL22 production through an alternative NF-κB axis.

**Figure 4:**
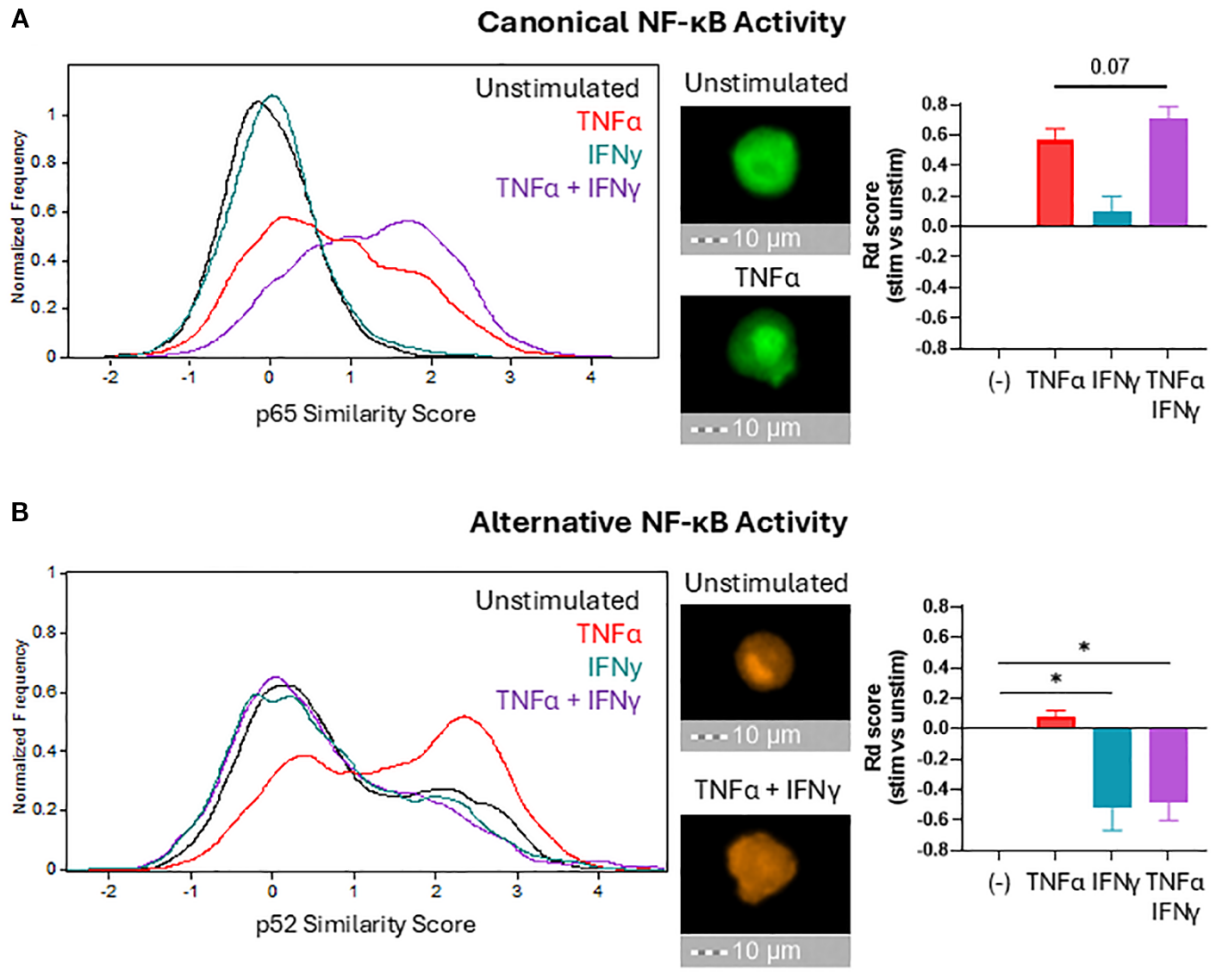
IFNγ selectively suppresses alternative NF-κB signaling. After 1 hour (**(A)** to visualize canonical NF-κB translocation) or 24 hours (**(B)** to visualize alternative NF-κB translocation) of stimulation in the indicated conditions, macrophages were fixed, permeabilized, and stained for NF-κB proteins p65 (canonical) and p52 (alternative). Nuclear localization of NF-κB proteins was quantified by imaging cytometry using median Similarity Score analysis (histograms) and normalized using the Fisher’s Discriminant ratio (bar graphs). Bar graphs present the mean +/- SEM of six independent experiments with different donors. Representative images for key conditions are displayed on the right side of the histograms (40X magnification). *p < 0.05.

Intriguingly, the impact of alternative NF-κB blockade on CCL22 production closely mimicked the effects of IFNγ, suggesting a mechanistic relation between IFNγ and alternative NF-κB signaling. To test the impact of IFNγ on NF-κB signaling, we used imaging cytometry to evaluate the nuclear translocation of the NF-κB transcription factors (p65 for canonical, and p52 for alternative) and determine their activation status. Because each NF-κB pathway is activated with different kinetics (canonical NF-κB is rapid but alternative NF-κB is slow) (48), the time points of 1 hour and 24 hours were chosen to evaluate the impact of TNFα and IFNγ on the activation of each pathway. As expected, TNFα activated canonical NF-κB and induced nuclear translocation of p65 (**Figure 4A**). IFNγ alone was insufficient to induce p65 nuclear translocation and marginally increased it when combined with TNFα. Baseline levels of nuclear p52 were already detectable in unstimulated macrophages, indicating baseline activation of alternative NF-κB signaling in myeloid cells (**Figure 4B**) consistent with their substantial CCL22 production at baseline (**Figure 3B**). IFNγ profoundly reduced the levels of nuclear p52, directly demonstrating a selective antagonism of alternative NF-κB signaling by IFNγ (**Figure 4B**).

Alternative NF-κB signaling is regulated by regulating protein levels of the NF-κB-Inducing Kinase (NIK) (49). NIK is ubiquitinated and degraded in resting cells, but pathway activation triggers the release of NIK from degradation and accumulation of NIK to induce nuclear translocation of the alternative NF-κB transcription factors (50). We examined if IFNγ regulates protein levels of NIK within macrophages to suppress alternative NF-κB signaling. Baseline macrophages exhibited background levels of alternative NF-κB signaling, and the NIK protein levels support this finding (**Figure 4B**, **Supplementary Figure S6**). However, we observed no changes in NIK protein levels beyond the standard background levels in IFNγ-stimulated macrophages, indicating IFNγ suppresses alternative NF-κB independently of NIK protein accumulation.

## Discussion

Our data demonstrate a novel mechanism in which IFNγ suppresses alternative NF-κB activity in human myeloid cells to selectively reduce production of CCL22 that recruit Tregs to the TME, thus allowing locally-activated CTLs to induce CCL5 and CXCL10 production and recruit additional functional CTLs without Tregs (**Supplementary Figure S7**). These findings demonstrate a novel role of alternative NF-κB signaling in regulating the balance of different classes of chemokines produced in the TME to control the character of immune cell infiltration.

Our data help explain the paradoxical tumor promoting and anti-tumor roles of the NF-κB system in cancer immunobiology and the regulation of tumor-associated chemokines. Previous therapeutic interventions targeting the NF-κB system have shown limited success, at least partially due to canonical NF-κB exhibiting both pro-tumor and anti-tumor functions (25). Production of both Treg-attracting chemokine CCL22 and CTL-attracting chemokines CCL5 and CXCL10 require canonical NF-κB signaling (12, 17), which limit the effectiveness of direct canonical NF-κB-targeting therapeutics. These observations provide rationale for exploring new options to enhance the desired effects but limit the detrimental effects of canonical NF-κB signaling.

Our data are aligned with the role of canonical NF-κB signaling as critical but insufficient alone to induce high levels of either desirable CTL attractants or detrimental Treg attractants. Rather, production of these factors requires both canonical NF-κB signaling and different co-factors. Optimal CXCL10 production requires additional IFNγ signaling as a co-factor, while CCL22 production requires the additional involvement of alternative NF-κB (**Supplementary Figure 5**). These considerations highlight the role of canonical NF-κB as a central factor for mobilizing inflammation in the TME (30) but dependent on other factors to regulate the specificity of its actions. Such specificity can involve interference with intracellular signaling pathways as demonstrated by the suppression of alternative NF-κB signaling by IFNγ (**Figure 4B**) and the suppression of IFNγ effector function in DCs by PGE2 (51).

Our previous *ex vivo* study (12) demonstrated that high baseline levels of canonical NF-κB signaling in the TME permit the CKM regimen (double-stranded RNA and IFNα) to selectively target tumor instead of healthy tissue. Accordingly, our recently completed clinical trials demonstrated that selective enhancement of CTL attraction to tumor tissues can be achieved not only by intratumoral (21, 22) but also systemic application (18, 23) of the CKM. Considering these results, it remains to be tested if enhanced levels of alternative NF-κB signaling in the TME may be limiting the positive effects of CKM and whether inhibition of alternative NF-κB signaling can prolong the effects of CKM to achieve more durable immune responses. Small molecule inhibitors targeting alternative NF-κB signaling have shown efficacy and safety in murine models (52). Administration of such drugs prior to CKM treatment could sensitize the TME and enhance CKM-mediated reprogramming of chemokine production and improve recruitment of CTLs.

Since the balance between canonical and alternative NF-κB signaling and the resulting chemokine patterns regulate multiple aspects of cancer cell and TME biology, our data imply the activation status of T cells in the TME is relevant to both their killing of cancer cells and to their TME remodeling functions. In addition to directly killing target cells within the TME, the production of effector molecules TNFα and IFNγ by activated T cells modulate immune cell recruitment within the TME by regulating this NF-κB balance – increasing the canonical while decreasing the alternative NF-κB activities. Since TNFα and IFNγ can be produced by both cytolytic and non-cytolytic CD8^+^ T cells including effector and memory cells (36, 38, 53), these data provide rationale for targeting tumor-resident non-effector CD8^+^ T cells to induce local production of CTL attractants and enhance intratumoral entry of more effective CTLs during adoptive cell therapy (ACT) or other forms of cancer therapy. They also raise the possibility of CTL involvement in the regulation of additional aspects of cancer cell biology such as proliferation, resistance to treatment-induced apoptosis, and metastatic potential, which all involve NF-κB. Although our current study did not test the ability of activated CTLs to recruit different subtypes of CD8^+^ T cells, our past studies showing the requirement for T cell activation to respond to CCR5- and CXCR3-binding chemokines suggest that CTLs favor recruitment of additional type-1 effector cells (CTLs, Th1, and NK cells) which all express CCR5 and CXCR3 (54).

Despite the ability of CTLs to promote COX2/PGE2-dependent suppression by MDSCs (38), our current data show that the induction of CTL-attracting chemokines was not accompanied by the induction of Treg-attracting chemokine CCL22 which is typically driven by the COX2/PGE2 axis (12, 17, 27, 28). Our findings help to explain this paradox by identifying a unique requirement for alternative NF-κB signaling for CCL22 production, the pathway which COX2 signaling enhances but IFNγ inhibits. This novel role of alternative NF-κB signaling in the production of CCL22 also explains the high baseline production of CCL22 by cultured macrophages since macrophages show high baseline levels of alternative NF-κB activation. IFNγ is largely considered a stimulatory factor that induces chemokine expression (CXCL9/10/11), but it can also suppress CCL3 and CCL4 production in peritoneal macrophages (55). Our current findings show that IFNγ blocks the production of suppressive chemokines but favors chemokines that recruit type-1 immune cells. The molecular mechanisms linking IFNγ signaling with the ability of alternative NF-κB transcription factors, RelB and p52, to interact with the CCL22 promoter remain unclear and are a topic of our upcoming studies, although we eliminated the involvement of decreased NIK protein (**Supplementary Figure S6**). Other mechanisms to be investigated are potential interference with p100 processing into the active p52 form, blocking NIK from activating the IKKα complex, or STAT-mediated suppression of IKKα activity (50). Our upcoming studies will also evaluate the interplay between TNFα, IFNγ, PGE2, and alternative NF-κB in regulating the balance between the pro- and anti-tumor functions of different myeloid cell types. Distinct myeloid populations are known to express different TNF receptors and may respond differently to TNFα and IFNγ (56). Additional differences may result from cell-specific unique epigenetic mechanisms modulating responses to TNFα and IFNγ (57–59). Since PGE2 signals through at least 4 different receptors and activates multiple pathways including cAMP, CREB, p38, and PI3K/Akt (60) their individual interactions with the alternative NF-κB signaling also remains to be established.

Interestingly, although the combination of TNFα and IFNγ could induce the production of CCL5 in ovarian cancer ascites cells or macrophages, their joint blockade did not abrogate induction of CCL5 in co-cultures of activated CD8^+^ T cells with ascites cells or myeloid cells. This result is consistent with our observation that in contrast to CXCL10 (which was produced predominantly by tumor-associated myeloid cells), a significant proportion of CCL5 originated from CD8^+^ T cells themselves in addition to the myeloid component by activated T cells. However, the combined blocking of TNFα and IFNγ eliminated the CD8^+^ T cell-enhanced ability of the TME to attract effector CTLs, indicating the CTL-produced CCL5 is not sufficient and highlighting the key role of myeloid cells in the additional CTL attraction.

In conclusion, our current study demonstrates a novel mechanism of suppression of alternative NF-κB by IFNγ that selectively promotes the expression of CTL-attracting chemokines and recruitment of CTLs by tumor-resident myeloid cells without the recruitment of Tregs. Our data identify the potential for manipulating alternative NF-κB signaling in the TME as a means of polarizing chemokine production and immune cell recruitment to favor recruitment of antitumor immune cells over suppressor cells. Given the critical requirement for tumor-infiltrating functional CTLs in durable immunity, our data provide rationale for combining alternative NF-κB modulation with immunotherapies to promote positive secondary immune effects, increase the influx of antitumor immune cells into the TME, and improve the durability of these responses.

## Data Availability

The raw data supporting the conclusions of this article will be made available by the authors, without undue reservation.

## References

[1] Hegde PS Chen DS. Top 10 challenges in cancer immunotherapy. Immunity. (2020) 52:17–35. doi: 10.1016/j.immuni.2019.12.011, PMID: 31940268

[2] Sharma P Hu-Lieskovan S Wargo JA Ribas A. Primary, adaptive, and acquired resistance to cancer immunotherapy. Cell. (2017) 168:707–23. doi: 10.1016/j.cell.2017.01.017, PMID: 28187290 PMC5391692

[3] Fridman WH Pages F Sautes-Fridman C Galon J. The immune contexture in human tumours: impact on clinical outcome. Nat Rev Cancer. (2012) 12:298–306. doi: 10.1038/nrc3245, PMID: 22419253

[4] Fridman WH Dieu-Nosjean MC Pages F Cremer I Damotte D Sautes-Fridman C. The immune microenvironment of human tumors: general significance and clinical impact. Cancer Microenviron. (2013) 6:117–22. doi: 10.1007/s12307-012-0124-9, PMID: 23108700 PMC3717061

[5] Gajewski TF. The next hurdle in cancer immunotherapy: overcoming the non-T-cell-inflamed tumor microenvironment. Semin Oncol. (2015) 42:663–71. doi: 10.1053/j.seminoncol.2015.05.011, PMID: 26320069 PMC4555998

[6] Curiel TJ Coukos G Zou L Alvarez X Cheng P Mottram P. Specific recruitment of regulatory T cells in ovarian carcinoma fosters immune privilege and predicts reduced survival. Nat Med. (2004) 10:942–9. doi: 10.1038/nm1093, PMID: 15322536

[7] Hou A Hou K Huang Q Lei Y Chen W. Targeting myeloid-derived suppressor cell, a promising strategy to overcome resistance to immune checkpoint inhibitors. Front Immunol. (2020) 11:783. doi: 10.3389/fimmu.2020.00783, PMID: 32508809 PMC7249937

[8] Oelkrug C Ramage JM. Enhancement of T cell recruitment and infiltration into tumours. Clin Exp Immunol. (2014) 178:1–8. doi: 10.1111/cei.12382, PMID: 24828133 PMC4360188

[9] Wu B Zhang B Li B Wu H Jiang M. Cold and hot tumors: from molecular mechanisms to targeted therapy. Signal Transduct Target Ther. (2024) 9:274. doi: 10.1038/s41392-024-01979-x, PMID: 39420203 PMC11491057

[10] Kohli K Pillarisetty VG Kim TS. Key chemokines direct migration of immune cells in solid tumors. Cancer Gene Ther. (2022) 29:10–21. doi: 10.1038/s41417-021-00303-x, PMID: 33603130 PMC8761573

[11] Musha H Ohtani H Mizoi T Kinouchi M Nakayama T Shiiba K. Selective infiltration of CCR5(+)CXCR3(+) T lymphocytes in human colorectal carcinoma. Int J Cancer. (2005) 116:949–56. doi: 10.1002/ijc.21135, PMID: 15856455

[12] Muthuswamy R Berk E Junecko BF Zeh HJ Zureikat AH Normolle D. NF-κB hyperactivation in tumor tissues allows tumor-selective reprogramming of the chemokine microenvironment to enhance the recruitment of cytolytic T effector cells. Cancer Res. (2012) 72:3735–43. doi: 10.1158/0008-5472.CAN-11-4136, PMID: 22593190 PMC3780565

[13] Jimenez-Sanchez A Memon D Pourpe S Veeraraghavan H Li Y Vargas HA. Heterogeneous tumor-immune microenvironments among differentially growing metastases in an ovarian cancer patient. Cell. (2017) 170:927–938 e920. doi: 10.1016/j.cell.2017.07.025, PMID: 28841418 PMC5589211

[14] Zou W. Regulatory T cells, tumour immunity and immunotherapy. Nat Rev Immunol. (2006) 6:295–307. doi: 10.1038/nri1806, PMID: 16557261

[15] Zou L Barnett B Safah H Larussa VF Evdemon-Hogan M Mottram P. Bone marrow is a reservoir for CD4+CD25+ regulatory T cells that traffic through CXCL12/CXCR4 signals. Cancer Res. (2004) 64:8451–5. doi: 10.1158/0008-5472.CAN-04-1987, PMID: 15548717

[16] Muthuswamy R Corman JM Dahl K Chatta GS Kalinski P. Functional reprogramming of human prostate cancer to promote local attraction of effector CD8(+) T cells. Prostate. (2016) 76:1095–105. doi: 10.1002/pros.23194, PMID: 27199259

[17] Theodoraki MN Yerneni S Sarkar SN Orr B Muthuswamy R Voyten J. Helicase-driven activation of NFκB-COX2 pathway mediates the immunosuppressive component of dsRNA-driven inflammation in the human tumor microenvironment. Cancer Res. (2018) 78:4292–302. doi: 10.1158/0008-5472.CAN-17-3985, PMID: 29853604 PMC6636317

[18] Gandhi S Opyrchal M Grimm MJ Slomba RT Kokolus KM Witkiewicz A. Systemic infusion of TLR3-ligand and IFN-alpha in patients with breast cancer reprograms local tumor microenvironments for selective CTL influx. J Immunother Cancer. (2023) 11(11). doi: 10.1136/jitc-2023-007381, PMID: 37963636 PMC10649898

[19] Obermajer N Urban J Wieckowski E Muthuswamy R Ravindranathan R Bartlett DL. Promoting the accumulation of tumor-specific T cells in tumor tissues by dendritic cell vaccines and chemokine-modulating agents. Nat Protoc. (2018) 13:335–57. doi: 10.1038/nprot.2017.130, PMID: 29345636

[20] Okada H Kalinski P Ueda R Hoji A Kohanbash G Donegan TE. Induction of CD8+ T-cell responses against novel glioma-associated antigen peptides and clinical activity by vaccinations with alpha-type 1 polarized dendritic cells and polyinosinic-polycytidylic acid stabilized by lysine and carboxymethylcellulose in patients with recurrent Malignant glioma. J Clin Oncol. (2011) 29:330–6. doi: 10.1200/JCO.2010.30.7744, PMID: 21149657 PMC3056467

[21] Orr B Mahdi H Fang Y Strange M Uygun I Rana M. Phase I trial combining chemokine-targeting with loco-regional chemoimmunotherapy for recurrent, platinum-sensitive ovarian cancer shows induction of CXCR3 ligands and markers of type 1 immunity. Clin Cancer Res. (2022) 28:2038–49. doi: 10.1158/1078-0432.CCR-21-3659, PMID: 35046055 PMC9106847

[22] Suarez Mora A Strange M Fang Y Uygun I Zhang L Tseng GC. Longitudinal modulation of loco-regional immunity in ovarian cancer patients receiving intraperitoneal chemotherapy. Cancers (Basel). (2022) 14(22). doi: 10.3390/cancers14225647, PMID: 36428740 PMC9688312

[23] Gandhi S Slomba RT Janes C Fitzpatrick V Miller J Attwood K. Systemic chemokine-modulatory regimen combined with neoadjuvant chemotherapy in patients with triple-negative breast cancer. J Immunother Cancer. (2024) 12(11). doi: 10.1136/jitc-2024-010058, PMID: 39542655 PMC11575314

[24] Geismann C Schafer H Gundlach JP Hauser C Egberts JH Schneider G. NF-κB dependent chemokine signaling in pancreatic cancer. Cancers (Basel). (2019) 11(10). doi: 10.3390/cancers11101445, PMID: 31561620 PMC6826905

[25] Yu H Lin L Zhang Z Zhang H Hu H. Targeting NF-κB pathway for the therapy of diseases: mechanism and clinical study. Signal Transduct Target Ther. (2020) 5:209. doi: 10.1038/s41392-020-00312-6, PMID: 32958760 PMC7506548

[26] Hayden MS Ghosh S. Shared principles in NF-κB signaling. Cell. (2008) 132:344–62. doi: 10.1016/j.cell.2008.01.020, PMID: 18267068

[27] Obermajer N Muthuswamy R Odunsi K Edwards RP Kalinski P. PGE(2)-induced CXCL12 production and CXCR4 expression controls the accumulation of human MDSCs in ovarian cancer environment. Cancer Res. (2011) 71:7463–70. doi: 10.1158/0008-5472.CAN-11-2449, PMID: 22025564 PMC4993027

[28] Muthuswamy R Urban J Lee JJ Reinhart TA Bartlett D Kalinski P. Ability of mature dendritic cells to interact with regulatory T cells is imprinted during maturation. Cancer Res. (2008) 68:5972–8. doi: 10.1158/0008-5472.CAN-07-6818, PMID: 18632653 PMC2905229

[29] Saccani S Pantano S Natoli G. Modulation of NF-κB activity by exchange of dimers. Mol Cell. (2003) 11:1563–74. doi: 10.1016/s1097-2765(03)00227-2, PMID: 12820969

[30] Liu T Zhang L Joo D Sun SC. NF-κB signaling in inflammation. Signal Transduct Target Ther. (2017) 2:17023. doi: 10.1038/sigtrans.2017.23, PMID: 29158945 PMC5661633

[31] Sun SC. The non-canonical NF-κB pathway in immunity and inflammation. Nat Rev Immunol. (2017) 17:545–58. doi: 10.1038/nri.2017.52, PMID: 28580957 PMC5753586

[32] Bai R Chen N Li L Du N Bai L Lv Z. Mechanisms of cancer resistance to immunotherapy. Front Oncol. (2020) 10:1290. doi: 10.3389/fonc.2020.01290, PMID: 32850400 PMC7425302

[33] Wherry EJ. T cell exhaustion. Nat Immunol. (2011) 12:492–9. doi: 10.1038/ni.2035, PMID: 21739672

[34] McLane LM Abdel-Hakeem MS Wherry EJ. CD8 T cell exhaustion during chronic viral infection and cancer. Annu Rev Immunol. (2019) 37:457–95. doi: 10.1146/annurev-immunol-041015-055318, PMID: 30676822

[35] Hoekstra ME Vijver SV Schumacher TN. Modulation of the tumor micro-environment by CD8(+) T cell-derived cytokines. Curr Opin Immunol. (2021) 69:65–71. doi: 10.1016/j.coi.2021.03.016, PMID: 33862306 PMC7610766

[36] Mailliard RB Egawa S Cai Q Kalinska A Bykovskaya SN Lotze MT. Complementary dendritic cell-activating function of CD8+ and CD4+ T cells: helper role of CD8+ T cells in the development of T helper type 1 responses. J Exp Med. (2002) 195:473–83. doi: 10.1084/jem.20011662, PMID: 11854360 PMC2193623

[37] Hoekstra ME Slagter M Urbanus J Toebes M Slingerland N de Rink I. Distinct spatiotemporal dynamics of CD8(+) T cell-derived cytokines in the tumor microenvironment. Cancer Cell. (2024) 42:157–167 e159. doi: 10.1016/j.ccell.2023.12.010, PMID: 38194914 PMC10783802

[38] Wong JL Obermajer N Odunsi K Edwards RP Kalinski P. Synergistic COX2 induction by IFNgamma and TNFalpha self-limits type-1 immunity in the human tumor microenvironment. Cancer Immunol Res. (2016) 4:303–11. doi: 10.1158/2326-6066.CIR-15-0157, PMID: 26817996 PMC4877699

[39] Muthuswamy R Wang L Pitteroff J Gingrich JR Kalinski P. Combination of IFNalpha and poly-I:C reprograms bladder cancer microenvironment for enhanced CTL attraction. J Immunother Cancer. (2015) 3:6. doi: 10.1186/s40425-015-0050-8, PMID: 25806105 PMC4371844

[40] Muthuswamy R Mueller-Berghaus J Haberkorn U Reinhart TA Schadendorf D Kalinski P. PGE(2) transiently enhances DC expression of CCR7 but inhibits the ability of DCs to produce CCL19 and attract naive T cells. Blood. (2010) 116:1454–9. doi: 10.1182/blood-2009-12-258038, PMID: 20498301 PMC2938836

[41] Peng D Kryczek I Nagarsheth N Zhao L Wei S Wang W. Epigenetic silencing of TH1-type chemokines shapes tumour immunity and immunotherapy. Nature. (2015) 527:249–53. doi: 10.1038/nature15520, PMID: 26503055 PMC4779053

[42] Nakayama T Hieshima K Nagakubo D Sato E Nakayama M Kawa K. Selective induction of Th2-attracting chemokines CCL17 and CCL22 in human B cells by latent membrane protein 1 of Epstein-Barr virus. J Virol. (2004) 78:1665–74. doi: 10.1128/jvi.78.4.1665-1674.2004, PMID: 14747532 PMC369498

[43] Dejardin E Droin NM Delhase M Haas E Cao Y Makris C. The lymphotoxin-beta receptor induces different patterns of gene expression via two NF-κB pathways. Immunity. (2002) 17:525–35. doi: 10.1016/s1074-7613(02)00423-5, PMID: 12387745

[44] Bonizzi G Bebien M Otero DC Johnson-Vroom KE Cao Y Vu D. Activation of IKKalpha target genes depends on recognition of specific κB binding sites by RelB:p52 dimers. EMBO J. (2004) 23:4202–10. doi: 10.1038/sj.emboj.7600391, PMID: 15470505 PMC524385

[45] Shin HM Kim MH Kim BH Jung SH Kim YS Park HJ. Inhibitory action of novel aromatic diamine compound on lipopolysaccharide-induced nuclear translocation of NF-κB without affecting IκB degradation. FEBS Lett. (2004) 571:50–4. doi: 10.1016/j.febslet.2004.06.056, PMID: 15280016

[46] Blaquiere N Castanedo GM Burch JD Berezhkovskiy LM Brightbill H Brown S. Scaffold-hopping approach to discover potent, selective, and efficacious inhibitors of NF-κB inducing kinase. J Med Chem. (2018) 61:6801–13. doi: 10.1021/acs.jmedchem.8b00678, PMID: 29940120

[47] Maguire O Collins C O'Loughlin K Miecznikowski J Minderman H. Quantifying nuclear p65 as a parameter for NF-κB activation: Correlation between ImageStream cytometry, microscopy, and Western blot. Cytometry A. (2011) 79:461–9. doi: 10.1002/cyto.a.21068, PMID: 21520400 PMC3140714

[48] Hayden MS Ghosh S. NF-κB in immunobiology. Cell Res. (2011) 21:223–44. doi: 10.1038/cr.2011.13, PMID: 21243012 PMC3193440

[49] Xiao G Harhaj EW Sun SC. NF-κB-inducing kinase regulates the processing of NF-κB2 p100. Mol Cell. (2001) 7:401–9. doi: 10.1016/s1097-2765(01)00187-3, PMID: 11239468

[50] Sun SC. Non-canonical NF-κB signaling pathway. Cell Res. (2011) 21:71–85. doi: 10.1038/cr.2010.177, PMID: 21173796 PMC3193406

[51] Vieira PL de Jong EC Wierenga EA Kapsenberg ML Kalinski P. Development of Th1-inducing capacity in myeloid dendritic cells requires environmental instruction. J Immunol. (2000) 164:4507–12. doi: 10.4049/jimmunol.164.9.4507, PMID: 10779751

[52] Brightbill HD Suto E Blaquiere N Ramamoorthi N Sujatha-Bhaskar S Gogol EB. NF-κB inducing kinase is a therapeutic target for systemic lupus erythematosus. Nat Commun. (2018) 9:179. doi: 10.1038/s41467-017-02672-0, PMID: 29330524 PMC5766581

[53] Watchmaker PB Urban JA Berk E Nakamura Y Mailliard RB Watkins SC. Memory CD8+ T cells protect dendritic cells from CTL killing. J Immunol. (2008) 180:3857–65. doi: 10.4049/jimmunol.180.6.3857, PMID: 18322193 PMC2905219

[54] Watchmaker PB Berk E Muthuswamy R Mailliard RB Urban JA Kirkwood JM. Independent regulation of chemokine responsiveness and cytolytic function versus CD8+ T cell expansion by dendritic cells. J Immunol. (2010) 184:591–7. doi: 10.4049/jimmunol.0902062, PMID: 20018619 PMC2922038

[55] Hodge-Dufour J Marino MW Horton MR Jungbluth A Burdick MD Strieter RM. Inhibition of interferon gamma induced interleukin 12 production: a potential mechanism for the anti-inflammatory activities of tumor necrosis factor. Proc Natl Acad Sci U.S.A. (1998) 95:13806–11. doi: 10.1073/pnas.95.23.13806, PMID: 9811882 PMC24904

[56] Atretkhany KN Gogoleva VS Drutskaya MS Nedospasov SA. Distinct modes of TNF signaling through its two receptors in health and disease. J Leukoc Biol. (2020) 107:893–905. doi: 10.1002/JLB.2MR0120-510R, PMID: 32083339

[57] Ivashkiv LB. IFNgamma: signalling, epigenetics and roles in immunity, metabolism, disease and cancer immunotherapy. Nat Rev Immunol. (2018) 18:545–58. doi: 10.1038/s41577-018-0029-z, PMID: 29921905 PMC6340644

[58] Lu Z Zou J Li S Topper MJ Tao Y Zhang H. Epigenetic therapy inhibits metastases by disrupting premetastatic niches. Nature. (2020) 579:284–90. doi: 10.1038/s41586-020-2054-x, PMID: 32103175 PMC8765085

[59] Veglia F Sanseviero E Gabrilovich DI. Myeloid-derived suppressor cells in the era of increasing myeloid cell diversity. Nat Rev Immunol. (2021) 21:485–98. doi: 10.1038/s41577-020-00490-y, PMID: 33526920 PMC7849958

[60] Kalinski P. Regulation of immune responses by prostaglandin E2. J Immunol. (2012) 188:21–8. doi: 10.4049/jimmunol.1101029, PMID: 22187483 PMC3249979

