# Supplementary figures and images for: IFNγ-mediated suppression of alternative NF-κB in tumor-resident myeloid cells promotes selective recruitment of cytotoxic but not regulatory T cells
Source: Front Immunol. 2025 Oct 9;16:1681777. doi: 10.3389/fimmu.2025.1681777 (PMC12546131; doi:10.3389/fimmu.2025.1681777)

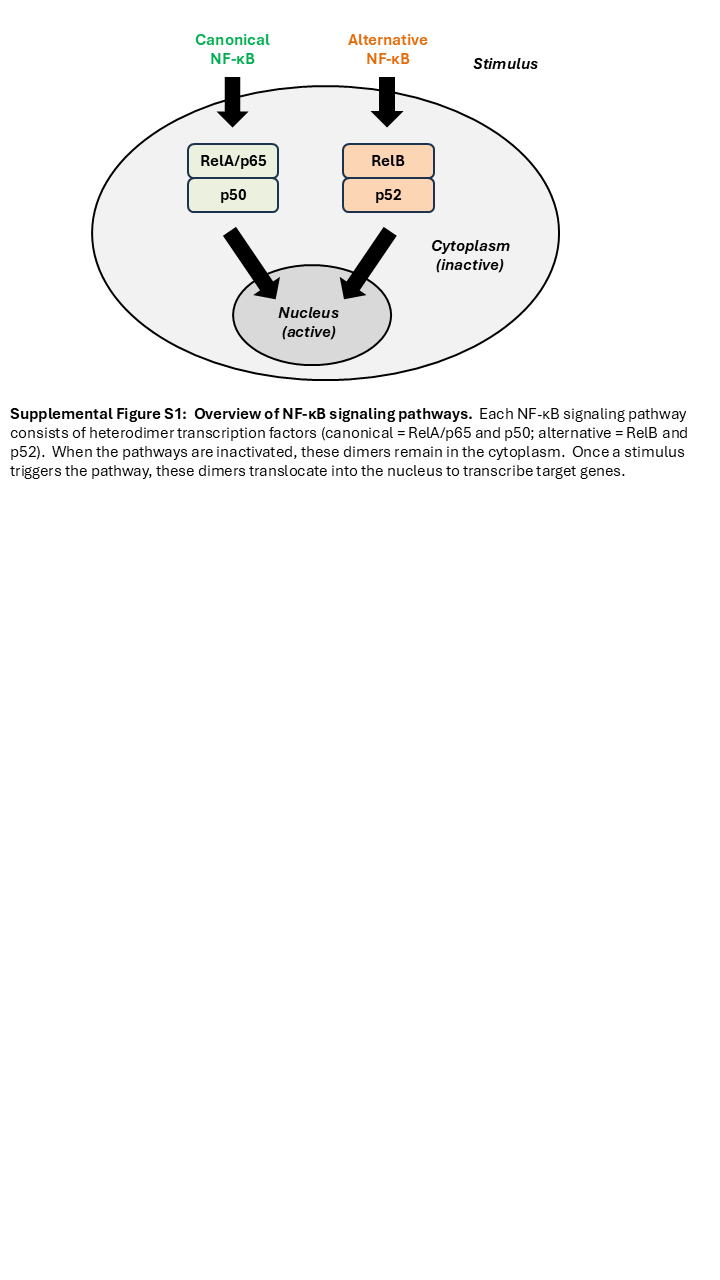

Supplement: Supplementary file 1 [file Image1.tif]

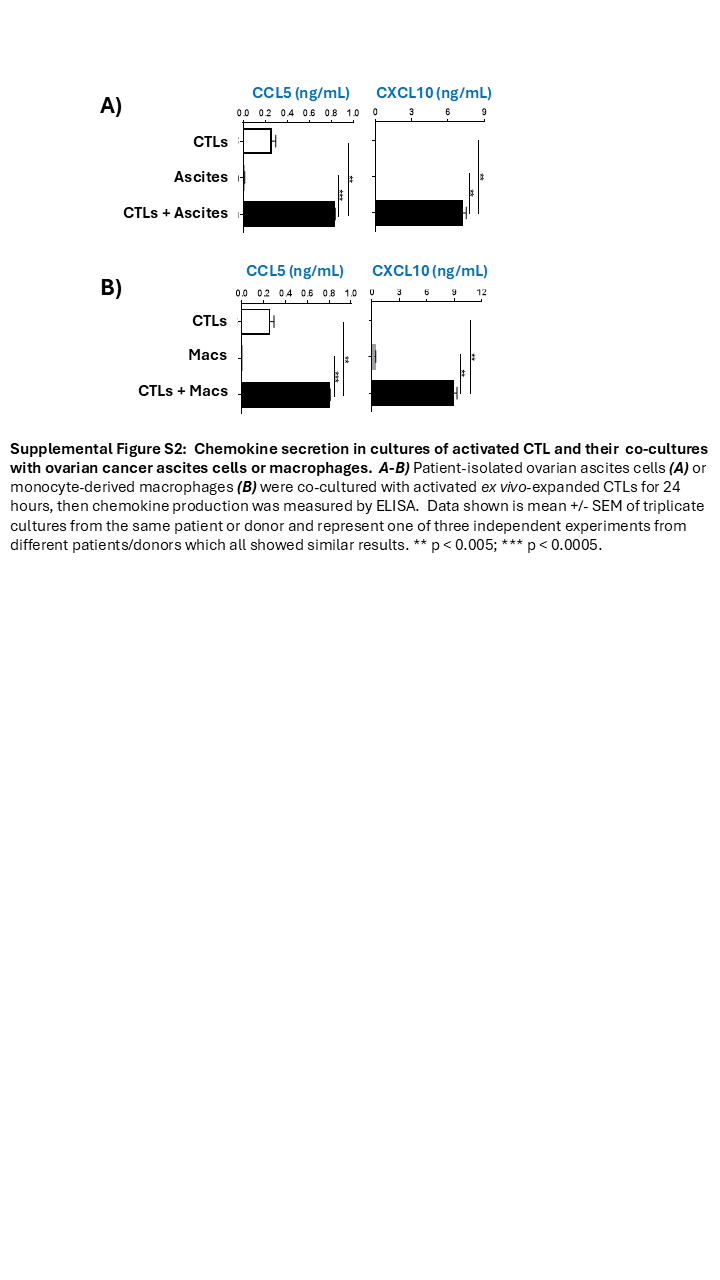

Supplement: Supplementary file 2 [file Image2.tif]

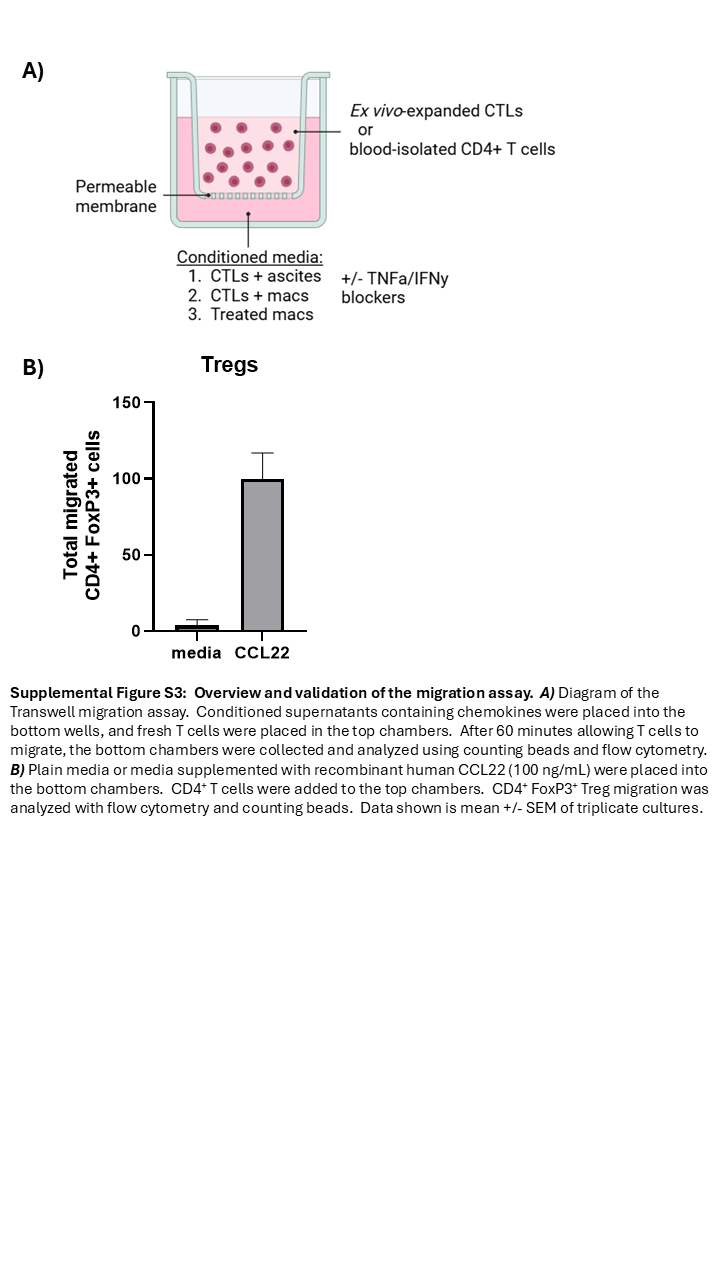

Supplement: Supplementary file 3 [file Image3.tif]

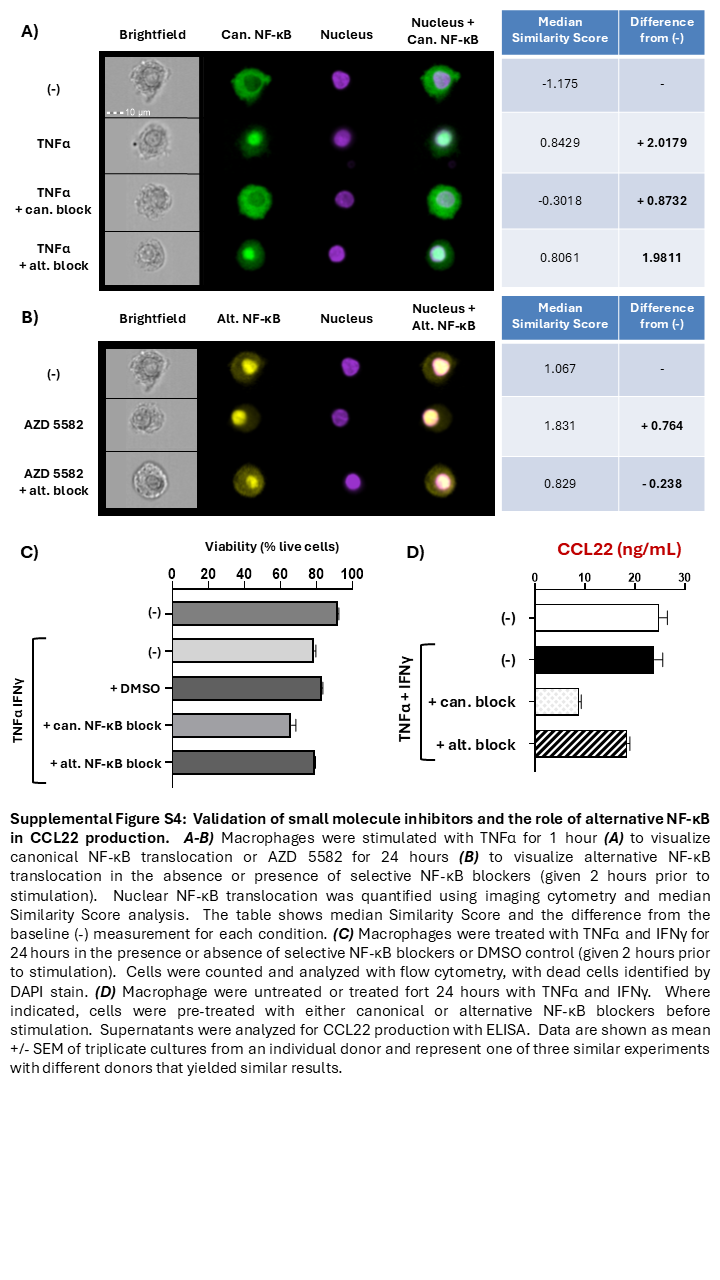

Supplement: Supplementary file 4 [file Image4.tif]

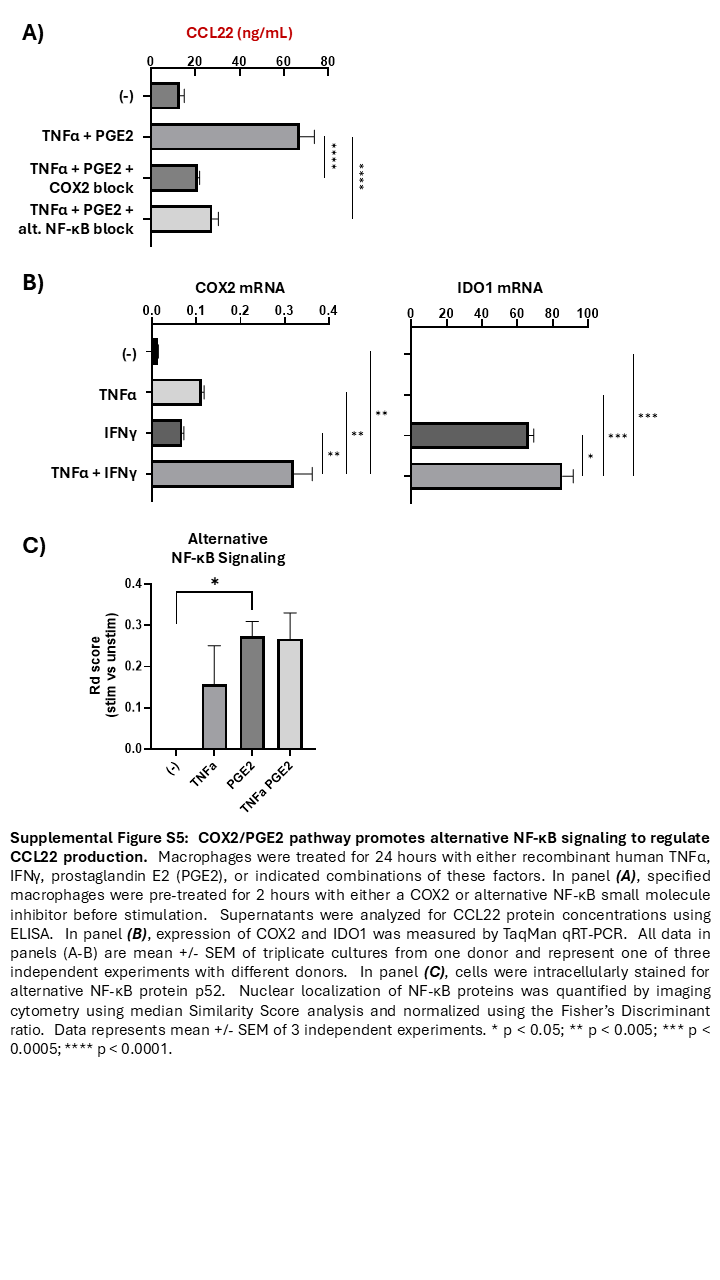

Supplement: Supplementary file 5 [file Image5.tif]

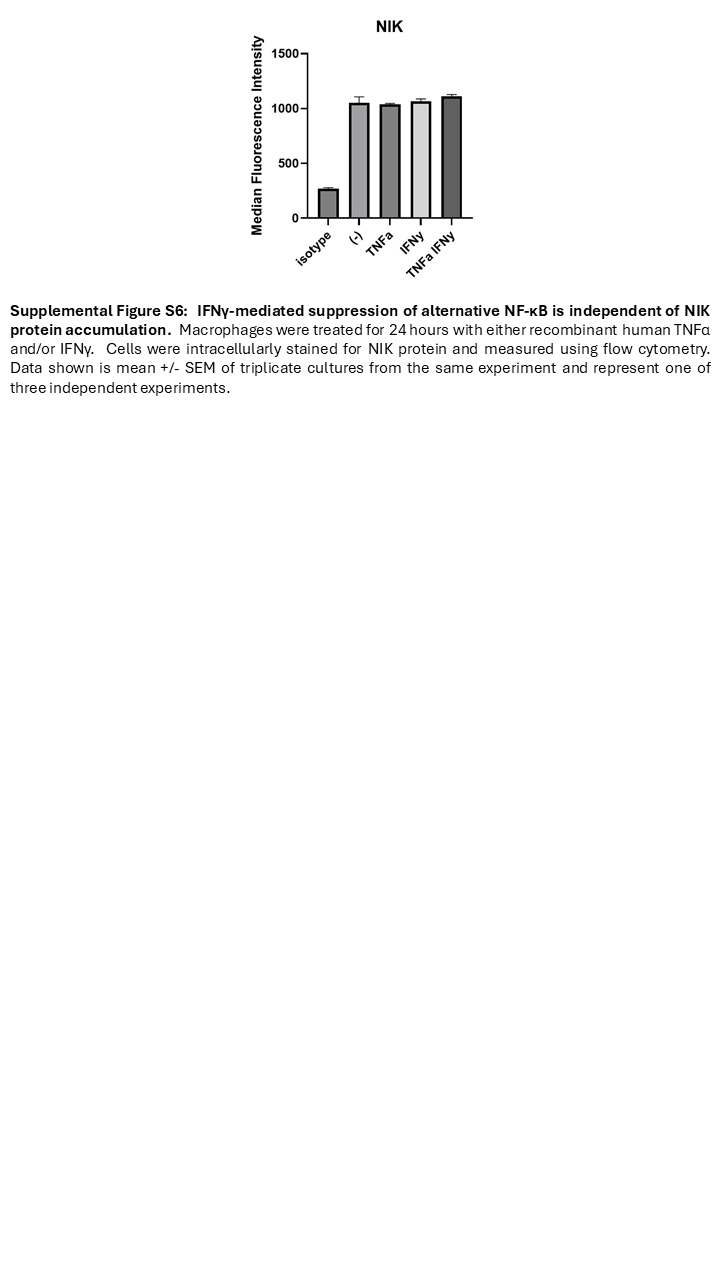

Supplement: Supplementary file 6 [file Image6.tif]

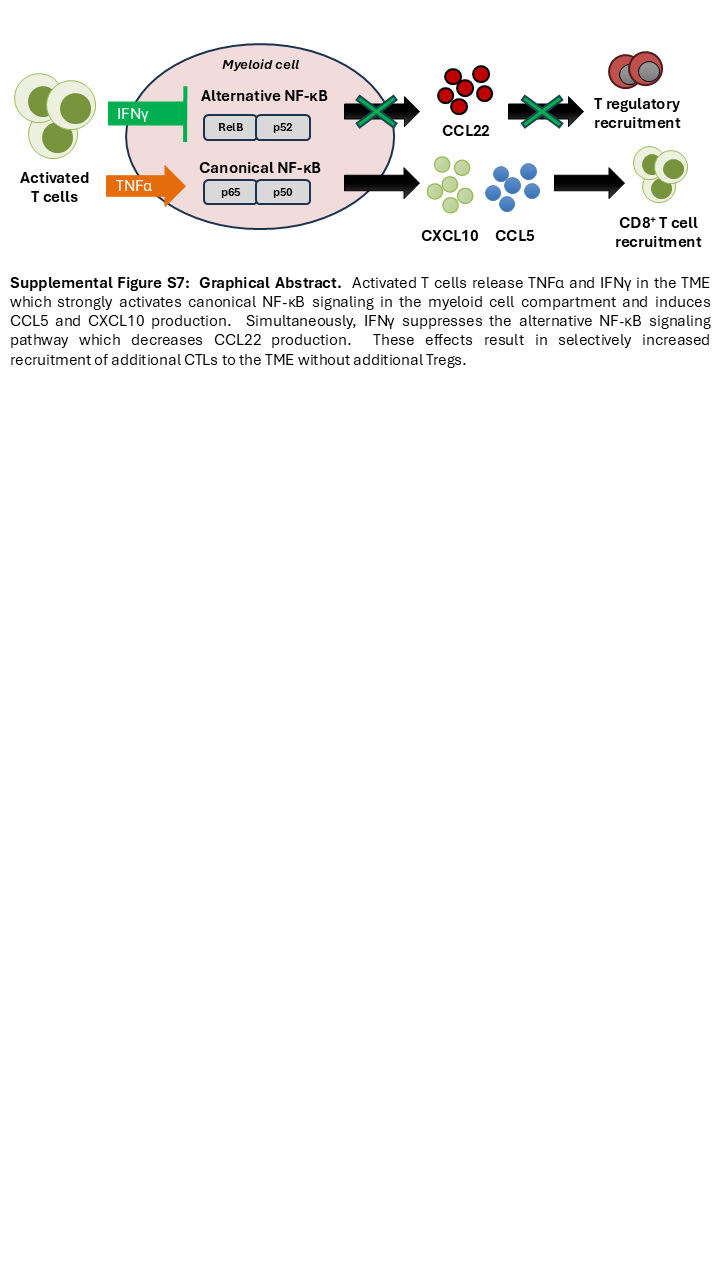

Supplement: Supplementary file 7 [file Image7.tif]
